# Evaluating the Impact of the Synar Program: Tobacco Access and Use among Youth in Mississippi, the South, and the U.S.

**DOI:** 10.3390/healthcare8010004

**Published:** 2019-12-22

**Authors:** Jerri S. Avery, John P. Bartkowski, Xiaohe Xu, Janelle Kohler, Melissa Mason

**Affiliations:** 1 Burton and Associates, Madison, MS 39110, USA; jerriavery@gmail.com; 2 Department of Sociology, University of Texas at San Antonio, San Antonio, TX 78249, USA; xiaohe.xu@utsa.edu (X.X.); mfmason0812@me.com (M.M.); 3 School of Public Administration, Sichuan University, Chengdu 610065, China; 4 Department of Psychology, University of Texas at San Antonio, San Antonio, TX 78249, USA; janelle.kohler@utsa.edu

**Keywords:** Synar, youth, commercial access, smoking, tobacco policy, diffusion of innovation, structuration, public health

## Abstract

(1) *Background*: This study examines the impact of Synar policy adoption on youth commercial access to tobacco products in Mississippi, the South, and the remaining U.S. The principal focus on youth commercial access is complemented by analyses of Synar’s impact on minors’ non-commercial access to tobacco and tobacco use patterns. Mississippi has been especially aggressive in implementing Synar, as evidenced by its unusually low retailer violation rates (RVRs). Synar, a mandatory, enforceable regulation meant to limit youth’s retail access to tobacco, was implemented nationwide in 1997. This study is governed by a combination of conceptual insights from a diffusion of health innovation perspective and structuration theory. (2) *Methods*: Repeated cross-sectional data from 1995 to 2011 from the CDC Youth Risk Behavior Survey are analyzed using a pre/post-implementation, quasi-experimental analytic strategy. Tobacco access and use in the pre-Synar era (1995–1997) are compared with two post-Synar periods (1999–2005 and 2007–2011), thereby highlighting diffusion effects related to this policy innovation within Mississippi, the South, and the remaining U.S. (3) *Results*: Analyses of temporal trends reveal that Mississippi and other study regions effectively restricted commercial access to tobacco. Positive outcomes associated with Synar adoption were observed several years after initial implementation, thus supporting a diffusion of innovation perspective. However, results also reveal that Mississippi youth were more inclined than their counterparts elsewhere to gain access to tobacco through non-commercial means after Synar implementation, and that declines in tobacco use among Mississippi youth were less robust than those observed elsewhere. Such variegated effects are in line with expectations linked to structuration theory. (4) *Conclusions*: Synar policy implementation has been generally effective at deterring youth access to tobacco and, in many cases, has yielded declines in tobacco use. However, there is no evidence that especially aggressive retailer compliance checks in Mississippi have yielded distinctive benefits for youth in this state.

## 1. Introduction

Tobacco use among adolescents has been a persistent problem in the United States and in the state of Mississippi [1]. In the wake of rising concerns about the human and financial costs of smoking-related illnesses, youth access restrictions have been implemented nationwide via partnerships between the federal government and state governments. The Synar Amendment was initially passed as federal legislation in 1992 [2,3]. The Synar Amendment requires all states to pass and enforce laws that prohibit the sale of tobacco to individuals under the age of 18. Synar requires retailers to verify a customer’s age prior to any tobacco sales, typically through the customer’s presentation of a valid driver’s license featuring the person’s date of birth. This practice is generally referred to as carding the customer. Retailers are required to refuse to sell tobacco to persons under the age of 18. Retailers must also store tobacco products in a secure location, typically a locked cabinet that is not directly accessible to customers. Therefore, customers must request tobacco products at the point of sale and such products must be retrieved by a retail representative (often, a manager) who has keys to the locked cabinet in which tobacco products are securely stored. For their part, states are charged with conducting stings (unannounced compliance checks) of retail establishments to ensure that they card customers who request tobacco products, refuse the sale of tobacco to minors, and store tobacco products securely under lock and key. Failure to comply with these requirements can lead to the retailer being fined or ultimately losing its license to sell tobacco products. Given the significance of these changes, momentum for Synar implementation was lacking for some time. It was not until 1996 that the Substance Abuse and Mental Health Services Administration (SAMHSA) began mandating that states enforce Synar through regular retailer compliance checks and report annually on the results of these efforts [2]. Enforcement efforts took some time to be organized and conducted, so serious Synar implementation on the state level did not begin in earnest until roughly 1997 and 1998.

Many studies have examined the contours and effects of tobacco control efforts directed at youth [4,5,6], and several have considered the influence of Synar implementation [7,8]. Tobacco control initiatives and, more specifically, Synar implementation have been shown to reduce smoking quite significantly among minors. Therefore, public health strategies designed to promote tobacco cessation among minors have gained considerable momentum. One especially relevant nationwide study conducted in the U.S. indicated that state-level Synar compliance accounted for a more than 20% reduction in the odds of youth smoking [7]. Tobacco prices were also shown to be a major influence on youth smoking, such that higher prices deterred minors’ tobacco usage. Such research is part of a larger body of scholarship on youth tobacco consumption that has also pinpointed a wide range of predictors (e.g., family cohesion) and consequences (e.g., impulsivity) associated with youth tobacco use [9,10]. Similar patterns have been explored not only within the U.S., but elsewhere around the world [7,11,12]. 

Commercial establishments’ noncompliance with the Synar Amendment is reported in the form of a retailer violation rate (RVR), which is the percentage of inspected outlets in a specified jurisdiction that fail to comply with legal obligations imposed by Synar [7,8,13]. Those obligations include having a current license to sell tobacco products, maintaining strict control over tobacco inventories by securing them in an area not accessible to customers, carding all customers requesting such products, and refusal to sell tobacco products to minors. In response to the Synar Amendment, the Mississippi Department of Mental Health, in collaboration with the Mississippi Office of the Attorney General, aggressively implemented a program in 1997 to reduce and monitor commercial youth access to tobacco and tobacco products [14]. Under this program, commercial establishments that sell tobacco are randomly tested to determine whether or not they comply with laws governing retail tobacco licensure, inventory control, and proper sales practices. Previous research reveals that retailer violation rates can vary among states, communities, and types of retailers; moreover, in some locales, violations can be quite common even well after the passage of Synar [7,8]. Such findings suggest that additional attention is needed to consider how Synar effectiveness might vary by geographical locale and over time [8]. With these considerations in mind, our study examines if Mississippi’s aggressive implementation of the Synar Amendment led to a distinctively robust reduction of youth tobacco access and use when compared with other Southern states and non-Southern states. Rather than using RVRs to examine this issue, survey data in which youth indicate the commercial availability of tobacco, along with minors’ non-commercial access to tobacco and self-reported tobacco use, are enlisted to conduct this study. RVRs can be subject to a range of factors (actual retailer compliance, the frequency and protocol of stings, the degree of enforcement after stings, etc.). Survey data are a more valid indicator of actual youth access to tobacco and their use of this substance.

In 2011, Mississippi continued to surpass significantly the national average of cigarette consumption [15]. The Synar Amendment stipulated that Mississippi and other states successfully reduce the ability of youth to purchase tobacco products or risk losing a substantial portion of block grant funds provided to states by the federal government. Block grant funds support substance abuse services in Mississippi. Thus, Mississippi’s inability to diminish youth commercial access to tobacco would have had larger ramifications within the state by undermining overall drug prevention efforts underwritten solely by block grant funds. SAMHSA is the federal agency tasked with determining state-level enforcement of Synar mandates. States are charged with monitoring commercial establishments that sell tobacco to ensure their compliance with laws regarding the placement and sales of tobacco products. Thus, states must ensure that consumers can only gain access to tobacco products through a licensed commercial retailer who is required to check identification and verify the age of would-be purchasers at the point of sale [16]. States conduct periodic sting operations in tobacco retail establishments to test retailer compliance with Synar mandates and must then report noncompliance in the form of RVRs to SAMHSA on a regular basis.

**Figure 1:**
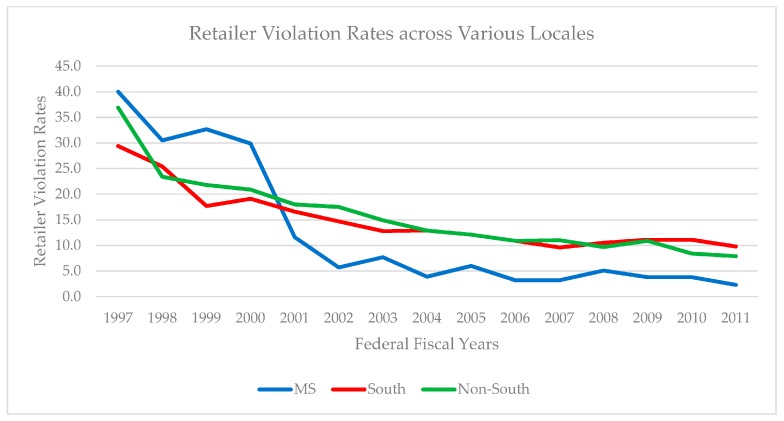
Retailer Violation Rates for Mississippi, the South, and non-South. RVRs are weighted by population.

Before proceeding, some additional contextual information is warranted. RVR comparisons between Mississippi, other Southern states (Mississippi’s peers), and non-Southern states (the remainder of the U.S.) are featured in Table 1 (annual statistics) and Figure 1 (trend lines). These data provide a helpful context and compelling rationale for our investigation. In 1997, the South collectively exhibited the lowest RVR and Mississippi exhibited the highest RVR. Non-Southern states fell between these two statistical poles. RVRs for Mississippi, other Southern states, and non-Southern states generally trended downward from 1998–2000 but Mississippi’s relatively high RVRs persisted. However, in 2001, the rank-order of RVRs across these three groups changed dramatically. In that year, Mississippi’s RVR plummeted to 11.6 percent, which was less than half of its 29.9 percent RVR in 2000. Mississippi’s 2001 RVR was 6.4 percent lower than that for non-Southern states, and 5 percent lower than other Southern states. Mississippi’s distinctively low RVRs were maintained through 2011. These patterns provide preliminary evidence that considerable time was required for Synar to be diffused effectively while also indicating that diffusion might be uneven across implementation locales.

What does the broader research literature indicate concerning Synar effectiveness? A handful of previous studies have evaluated the impact of Synar implementation, and several point to some measure of success concerning the impact of this policy [6,7,17,18,19,20]. One detailed audit of state and federal documents related to Synar revealed a universal adoption of laws to restrict youth tobacco access and near universal enforcement of such laws, with the latter conclusion supported by a marked decline in RVRs in the early 2000s [21]. However, there are also indications that the lack of a good faith effort in many states, combined with the Department of Health and Human Services’ decision to not require states to enforce their laws by penalizing lawbreakers, significantly slowed the implementation of Synar [21]. Other inquiries have revealed program effectiveness in terms of state-level RVRs [22], although some caution is warranted in using RVRs as a measure of success given disparities in sting implementation and policy enforcement across states. Taken together, these studies provide some evidence of Synar’s short-term effectiveness at the retailer distribution level while hinting that success may be uneven, given differences in implementation. And one study of long-term effects does indicate that stringent retailer compliance reduced youth tobacco usage [7].

Consumer-level effects of Synar implementation, including youth commercial access to tobacco and tobacco use, are arguably the litmus test of the program’s effectiveness and have yet to be investigated as we have proposed here. We examine this issue using several waves of Youth Risk Behavior Survey (YRBS) data principally grounded in a diffusion of innovation theoretical model [26,27,28]. Briefly, the diffusion model presumes that the intended aim of a healthcare policy will be more evident long after that policy has been initially adopted when compared with immediately after its implementation. In essence, health policy innovations require considerable time before achieving their intended results. Some policies result in increasing impact over time as greater numbers of people are affected by the policy. Increasingly positive effects associated with the long-term implementation of healthcare innovations can also be attributed to various possible mechanisms associated with the diffusion process. While specific implementation improvement mechanisms are difficult to test empirically (and we cannot do so in this study), two such possibilities are directly relevant to Synar. First, states may ultimately identify and adopt improved regulatory controls that are elusive upon initial implementation. Improved state-level regulatory control practices may be used to “iron out” implementation “wrinkles” quite pragmatically. For example, states may become more effective at conducting Synar-based stings of tobacco retailers that serve as valid compliance checks that are unanticipated by retailers. Furthermore, fines that are imposed for retailer non-compliance might be adjusted over time to maximize compliance at the point of sale. If states collect data about control and compliance processes, data-driven decision-making (DDDM) techniques can be enlisted to foster such implementation gains. But, of course, DDDM requires time associated with the collection and analysis of data, as well as the integration of data-driven practical refinements. Second, where tobacco retailers themselves are concerned, implementation improvements may become increasingly influential over time as such commercial establishments determine the most effective training and internal compliance techniques to ensure that their employees conduct customer identification checks (“carding”) of those seeking to purchase tobacco products. Moreover, because Synar requires retailers to secure tobacco products under lock and key so that only employees can access these goods upon valid customer request, it is reasonable to assume that infrastructure and procedural changes related to this facet of compliance may require time to be instituted and routinized. Synar implementation is anticipated to follow this trajectory based on the diffusion of health innovation model. Research on various public health programs supports this argument, such that health policy innovations are often more effective several years after they have been initiated because of the time needed to diffuse new policies and practices throughout the social environment.

Our study also aims to augment existing diffusion of innovation models in two notable ways. First, structuration theory, which originated with sociologist Anthony Giddens but has also been used to study systemic health promotion efforts, offers a compelling argument against presuming that diffusion is a deterministic, uniform, or unilinear process [29,30,31,32]. Structuration theory contends that the introduction of novel social structures, including innovative legal policies such as Synar, may be at once fruitful and limited in their ability to direct courses of conduct among the people at whom they are aimed. Structures influence action, but do not determine conduct. Why? Structures are often marked by gaps and fissures—what Giddens [33] calls “structural contradictions” or countertendencies—that can be leveraged or exploited by groups of social actors who use their agency to resist intended changes. From a structuration theory perspective, a social structure is composed of the interplay between rules (e.g., habits, norms, laws) and resources (e.g., monetary funds, human effort). Giddens describes this interplay between rules and resources as a “duality of structure,” and this concept proves quite helpful for thinking about Synar. As a legal structure, Synar is a policy-based environmental strategy that introduces new rules (storing tobacco securely, carding customers, refusing to sell tobacco to minors) and resource allocations (noncompliance penalties) into the point of sale for young people seeking to purchase tobacco products. Synar is designed to redirect en masse the conduct of young people based on retailers verifying the age of would-be tobacco consumers, thereby deterring minors who might attempt to purchase tobacco products by instituting significant financial and licensure penalties for such transactions. Yet, as with any structure, the Synar policy has limitations. An excellent example of a structural fissure embedded in Synar is its focus on young people’s *commercial* access to tobacco. Laws that curb youth commercial access to tobacco leave open the possibility of young people using other *non-commercial* means to secure tobacco. Thus, as a legal structure, Synar does not simply delimit action by prohibiting young people’s purchase of tobacco through commercial means. For some, it may enhance the attractiveness of alternative courses of conduct, including youth enlisting other adults (friends, older siblings, confidants) who will provide these minors with tobacco that the adults purchase on behalf of youth or lend to them. In the language of structuration theory, these gray market “workaround” transactions are a form of *agency* intentionally aimed at sidestepping the retailer restrictions imposed by Synar. In short, structural innovations may incite creative variations in social practice at the grassroots level. Therefore, a careful consideration of Synar’s effectiveness must not only attend to young people’s commercial access to tobacco, but should examine alternative trends in non-commercial access as well.

Second, for any policy innovation, it is important to distinguish between first-order and second-order effects, sometimes described as “upstream” and “downstream” factors, respectively [34,35]. The stated intent of Synar is to limit or even eliminate young people’s commercial access to tobacco. Thus, a study designed to evaluate Synar effectiveness must consider the policy’s first-order effects, that is, the extent to which youth commercial access is curbed by Synar adoption while also accounting for non-commercial alternatives. Yet, the most critical aim of Synar is not simply to deter young people’s ability to secure tobacco products. The policy’s ultimate objective is found in what might be called its second-order or “downstream” effect, namely, to deter and thereby reduce youth tobacco use. Hence, limiting access is an “upstream” means to achieve a larger “downstream” goal. For this reason, the present study examines not only tobacco access (a first-order effect of Synar), but also presents the results of an additional round of data analyses on youth tobacco use (a second-order effect of Synar implementation).

To what degree has diminished tobacco availability been diffused among Mississippi youth over time as a result of the implementation of the Synar regulation? How do these patterns compare with those exhibited elsewhere in the nation? Moreover, how does youth tobacco access compare with tobacco use trends among minors? The general expectation is that prolonged implementation of Synar will promote more restricted tobacco access among Mississippi youth and their peers elsewhere. And these first-order effects in more restricted tobacco access are anticipated to yield second-order consequences, namely, reductions in youth tobacco use. To address the foregoing research questions, the following hypotheses are developed and tested in the present study.

**Hypothesis** **1(H_1_).** *Mississippi youth will report diminished commercial access to tobacco as a result of the implementation of the Synar policy.*

**Hypothesis** **2(H_2_).** *Greater reductions in youth commercial access to tobacco will be observed long after Synar’s adoption (late post-implementation) rather than shortly after its adoption (early post-implementation).*

**Hypothesis** **3(H_3_).** *Greater reductions in youth commercial access to tobacco will be observed for Mississippi than for quasi-control groups (i.e., Southern and non-Southern states other than Mississippi).*

**Hypothesis** **4(H_4_).** *Greater reductions in youth tobacco use will be observed for Mississippi over the course of the study period in comparison with quasi-control groups (i.e., Southern and non-Southern states).*

## 2. Materials and Methods 

Data for this study are drawn from the national Youth Risk Behavior Survey (YRBS), a standardized biennial survey of middle and high school students in the U.S. developed by CDC. The YRBS is designed to determine the prevalence of high-risk behaviors among students in grades 6–12, and aims to assess how these behaviors increase, decrease, or remain stable over time [1]. This study utilizes high school students’ data from the YRBS that have been available since 1991. The target population is all public and private high school students in the fifty states and the District of Columbia. Even though Mississippi began participating in the survey in 1993, commercial access data weren’t available until 1995. As a result, when investigating youth commercial access to tobacco, this study includes 12,827 high school respondents from Mississippi, 56,730 high school respondents from other Southern states (defined by Census region, excluding Mississippi), and 71,077 high school respondents from non-Southern states, with a total of 140,634 high school respondents from 1995 to 2011. Because the national YRBS uses a three-stage cluster sampling design to obtain a nationally representative sample of students in grades 9–12 in the U.S., this study corrects the design effects with weights wherever possible. The national YRBS provides a sufficient sample size to produce estimates that are accurate within ±5% at a 95 percent confidence interval [36]. More recent YRBS data are not relevant for this study because (1) they move too far past the initial point of Synar implementation and (2) more recent waves are not considered representative within Mississippi, a key study region.

### 2.1. Dependent Variables

The principal dependent variable examined in this study consists of youth commercial access to tobacco. For reasons described above, a second round of analyses is also conducted with youth tobacco use serving as a dependent variable. The YRBS inquires about young people’s usual means of securing cigarettes (the most common form of youth consumption during the study period), and is operationalized as follows: “During the past 30 days, how did you usually get your own cigarettes?” There are eight response categories: 1 = I did not smoke cigarettes during the past 30 days, 2 = I bought them in a store such as a convenience store, supermarket, discount store, or gas station, 3 = I bought them from a vending machine, 4 = I gave someone else money to buy them for me, 5 = I borrowed (or bummed) them from someone else, 6 = A person 18 years old or older gave them to me, 7 = I took them from a store or family member, and 8 = I got them some other way. This variable was dummy-coded into a new variable with 1 = commercial access (purchased from a retailer or from a vending machine) and 0 = otherwise. Among original response options, response 1 is retained and placed in the “otherwise” category, because effective Synar implementation aims to limit commercial access and thereby lead to more prevalent abstinence from tobacco. Response 7 reflects theft of cigarettes from a store or a family member, not obtaining them through a lawful commercial transaction. It is placed in the “otherwise” category for that reason. A key goal of this study on commercial access is to distinguish between retail tobacco acquisition (coded 1) and all other possible outcomes (coded 0).

Youth tobacco use is operationalized by a survey question inquiring about respondents’ past 30-day cigarette smoking: “During the past 30 days, on how many days did you smoke cigarettes?” The YRBS provides seven response categories, coded as follows: 1 = zero days, 2 = one or two days, 3 = three to five days, 4 = six to nine days, 5 = 10 to 19 days, 6 = 20 to 29 days, and 7 = all 30 days. This indicator is treated as a metric variable by assuming an underlying continuum of days of cigarette smoking.

### 2.2. Independent Variable

The key independent variable for this investigation is *time*. With reduced youth access to tobacco and tobacco use hypothesized to occur, statistical comparisons of trend measures are rendered with data collected from 1995 to 2011. Time is the independent variable in the study because diffusion theory predicts that the desirable effects of the policy implementation would increase in magnitude over time. We compare results over three distinct study time periods: Synar pre-implementation (1995–1997), early post-implementation (1999–2005), and late post-implementation (2007–2011).

### 2.3. Control Variables

Socio-demographic characteristics available in the YRBS are controlled (e.g., age, gender, and race–ethnicity). Although the cost of tobacco in Mississippi cannot be controlled, it is worth noting that its price increased dramatically in 2009 (an approximately fifty cent increase per pack of cigarettes, cigars, and smokeless tobacco). Still, this factor would not be expected to affect retail access to youth—our principal focus—in a marked fashion, since Synar targets the commercial availability of tobacco to this population. Price increases aim to reduce consumption among those who can legally acquire tobacco products. We concede that price increases may impact tobacco use among adults, which could then affect youth access and consumption if minors enlist adults to secure tobacco products on their behalf.

### 2.4. Analytic Strategies

To analyze youth commercial access to tobacco and related dependent variables before and after Synar implementation, YRBS data were pooled and stacked across years. Dummy variables were created to distinguish Synar pre-implementation (1995–1997), early post-implementation (1999–2005), and late post-implementation (2007–2011) with pre-implementation serving as the reference. Adjusted proportions were generated for each dependent variable using the binary logistic regression procedure in SPSS. For this part of the data analysis, the binary logistic regression model was specified as Log[*p*(x)/1 − *p*(x)] = *α* + *β*_1_Age + *β*_2_Male + *β*_3_Black + *β*_4_Hispanic + *β*_5_Other Race. Using this model, the adjusted proportions were calculated to represent the percentages of youth reporting commercial access to tobacco net of statistical controls (i.e., age, gender, and race–ethnicity). This analytic strategy is akin to the analysis of covariance technique, where population means are estimated for the key categorical independent variables, controlling for covariates. Preliminary analyses indicated that all models were sufficiently powered to produce valid estimates. Because preliminary analyses revealed that age, gender, and race–ethnicity were significantly related to the dependent variables, adjusted proportions were necessary to account for these effects when examining differences in dependent variable values across time periods. A similar approach was used for past 30-day tobacco use, where the adjusted average values were estimated and reported while controlling for age, gender, and race–ethnicity.

Once the adjusted proportions were generated, they were plotted against survey years (i.e., 1995–2011) to examine temporal trends. Additional logistic regression models were developed to test separately for possible quasi-experimental effects in commercial access for Mississippi, Southern, and non-Southern states (H_1_ and H_2_). Formal statistical tests were conducted to determine if the differences in the quasi-experimental effects exhibited in Mississippi, Southern states, and non-Southern states were significantly different from one another (H_3_). The same methodological approach was utilized for examining temporal and spatial variations in youth tobacco use (H_4_).

## 3. Results

The principal aim of this study is to examine the impact of Mississippi’s Synar program on minors’ commercial access to tobacco. Ancillary aims entail analyzing variations in young people’s non-commercial access to tobacco and their past 30-day use of tobacco. With its distinctively low retailer violation rates (RVRs), Mississippi’s Synar program would be considered effective if significant declines in commercial tobacco access were observed among Mississippi youth. Additional evidence of effectiveness would be observed if Mississippi youth reported a significantly larger magnitude of decline in past 30-day tobacco use than their counterparts elsewhere.

Turning to the hypotheses that are tested in this study, it was first anticipated that Mississippi youth will report diminished commercial access to tobacco as a result of the Synar policy (H_1_). To investigate the degree of support for this hypothesis, trend data from YRBS were analyzed. Table 2 reports the adjusted proportions of commercial access and Table 3 shows the odds coefficients derived from a series of logistic regression models designed to test the first three hypotheses that govern our study. It must be noted that if the odds coefficients for the dummy-coded time period variables are greater than 1.0, then they would indicate greater commercial access in the two post-Synar periods as compared to the pre-Synar period. Likewise, if the odds coefficients are less than 1.0, then they would indicate diminished commercial access over time.

A careful examination of Table 2 reveals reductions in adjusted proportions of youth tobacco access over the three study periods in Mississippi, as well as in the South and the remaining U.S. composed of non-Southern states. Among Mississippi youth, the initial percentage of youth who reported having commercial access in 1995 was reduced from 18.66% to 5.75% by 2011, a more than 69 percent reduction ([18.66–5.75]/18.66). Although the magnitude of reduction in commercial access is greater in Mississippi (−69%) than in the remainder of the South (−63%), it is still less than reductions exhibited in the non-South (−73%). Moreover, the odds coefficients featured in the Mississippi column of Table 3 reveal statistically significant negative associations in both the early and late post-Synar eras (*p* < 0.001 for both variables) when compared to the pre-Synar period. Consequently, H_1′_s prediction of diminished commercial access to tobacco among Mississippi youth after Synar implementation is supported.

Hypothesis 2 predicted that salutary effects in youth tobacco access will be more pronounced long after Synar’s implementation (late post-implementation) than in the initial years after its adoption (early post-implementation). That is, more robust reductions are expected to be evident after this policy innovation has had sufficient time to have been diffused in the late post-implementation period. A comparison of the middle panel (early post-implementation) and the right panel (late post-implementation) of Table 2 is revealing. Noteworthy reductions in commercial access for Mississippi youth were observed in early post-implementation (1999–2003) (e.g., 6.74% access rate in 2001). However, those reductions were even more pronounced in late post-implementation (2007–2011), with the lowest rate observed in 2011 (5.75 percent access rate in 2011). Additional statistical tests were conducted comparing the reductions from pre-implementation to early post-implementation with reductions from pre-implementation to late post-implementation. As shown in Table 3, the odds of access in Mississippi were 52.6 [(0.474–1) × 100] percent and 68.1 [(0.319–1) × 100] percent lower, respectively, than those observed before Synar. Another round of statistical tests was conducted using regression parameter estimates. These tests indicate a robust, statistically significant difference (*p* = 0.001) based on the odds coefficients reported in Table 3. Therefore, Synar was much more effective in Mississippi during the late post-implementation period than the early post-implementation period. An additional statistical test with the same data used to generate the results featured in Table 3 was also conducted. This additional test compared the respective odds ratios for Mississippi’s early post-implementation era and late post-implementation era. This test revealed that the odds of access were 32.7% [(0.673–1) × 100] lower in the late post-implementation period than those in the early post-implementation period for Mississippi. This difference between early post-Synar implementation and late post-Synar implementation in Mississippi was highly significant (*p =* 0.001). Therefore, among Mississippi youth, H_2′_s prediction that the strongest impact of Synar would be observed after policy diffusion (i.e., late post-implementation) is strongly supported.

Hypothesis 3 anticipated that more salutary effects in youth tobacco access will be observed for Mississippi than for quasi-control groups (respectively, the South and the non-South). The adjusted proportions depicted in Table 2 reveal that reductions in youth tobacco access were evident among Mississippians as well as their Southern and non-Southern peers across the three study time periods. However, a visual inspection of these data alone cannot test H_3_.

**Figure 2:**
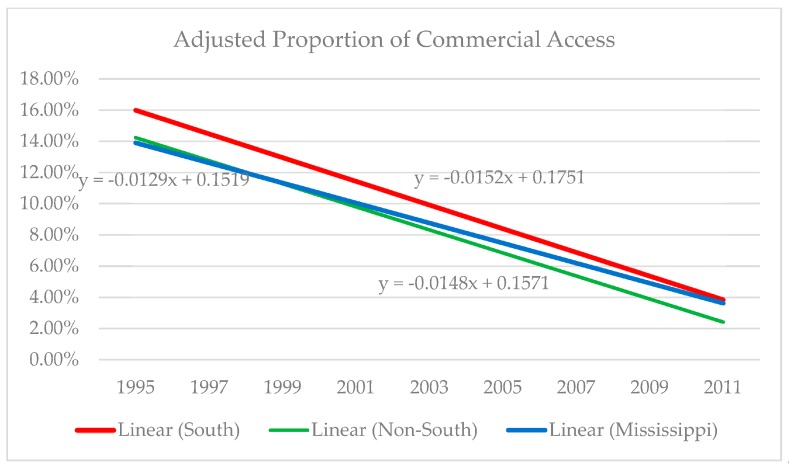
Adjusted proportion of commercial access. Log[*p*(x)/1 − *p*(x)] = a + *β*_1_Age + *β*_2_Gender + *β*_3_Black + *β*_4_Hispanic + *β*_5_Other Race.

Table 3 provides additional evidence of parity among Mississippi, Southern, and non-Southern youth, because all *p*-values in the relevant cells (early post-Synar and late post-Synar eras) are less than 0.001, which is the highest degree of statistical significance. These datapoints alone, however, do not verify that reductions among Mississippi youth were significantly similar to those of the comparison groups. Figure 2 depicts these respective reductions in graphic form and indicates roughly comparable slopes for each of the trend lines. Consequently, the Mississippi Synar program has been no more effective than its counterparts in the South and non-South in reducing commercial access. Therefore, H_3′_s prediction of distinctive effects for Mississippi is not supported. As a means of verifying the findings presented in Table 3, the interaction effects of the Synar era and study region were also estimated. These results are displayed in Table 4. These difference-in-differences statistical tests (interaction effects) were conducted to discern any possible youth tobacco access disparities evident among the odds ratios for the pre-Synar era (reference), early post-Synar era, and late post-Synar era across the three locales. The interaction effects featured in Table 4 reveal no significant results, thereby indicating that Synar was similarly effective in all three of our study locales in limiting young people’s commercial access to tobacco.

Table 5 reflects our effort to cross-check Synar effectiveness in light of alternative avenues through which young people might secure tobacco products. Consistent with the concepts of structural fissures (policy gaps) and agency (actors’ innovative social practices) from structuration theory, we examine the prospect of youth securing tobacco by (1) giving someone else money to purchase tobacco for them or (2) having someone older than 18 give them tobacco, both of which are response options on the YRBS. Consequently, we recoded the dependent variable so that these two options were coded as 1 and all other options were coded as 0. The findings are featured in Table 5. Significant distinctions are indeed evident for non-commercial access across our study locales. Increased non-commercial access to tobacco was evident in Mississippi in the early and late post-Synar eras, when compared with the pre-Synar period, thus suggesting that Mississippi youth pursued non-commercial means of securing tobacco once commercial access was restricted. Increasing reliance on non-commercial avenues for securing tobacco were inconsistent for youth in the South (significantly elevated for the early post-Synar period only) and were substantially reduced among youth in the non-South (significantly declined in the late post-Synar period only). These findings reveal important, geographically specific trends in non-commercial access, which is a noteworthy counterpart to consider when examining Synar’s impact. So, while Synar was equally effective in limiting commercial access to tobacco among youth, young people’s pursuit of alternative non-commercial avenues varied, with non-commercial means vigorously pursued by Mississippi youth. In short, the state that was among the nation’s leaders in retailer compliance with Synar unwittingly inspired its youth who were set on securing tobacco to utilize non-commercial means for doing so once commercial means were largely unavailable.

Echoing these geographically specific trends in non-commercial access are those evident for youth tobacco use, the second-order and ultimate objective of Synar. As revealed in Table 6, the adjusted average 30-day use of tobacco products (i.e., cigarette smoking) declined by 21% from 1993 to 2011 for youth in Mississippi. However, the magnitude of tobacco use reduction is greater in the South (24%) and non-South (27%) than in Mississippi during the same period of time. This finding suggests that past 30-day use of tobacco products was more difficult to change in Mississippi, possibly due to greater non-commercial avenues of access available to youth within this particular state. It is also possible that smaller magnitudes of change in Mississippi over time were influenced by a lower baseline average of tobacco use in 1993. Regardless, H_4′_s prediction of distinctively larger declines in tobacco use for Mississippi youth is not supported.

## 4. Discussion

Passed by Congress in 1992, the Synar Amendment is a cornerstone of public policy regarding tobacco control [2,3]. This policy targets youth commercial access to tobacco products by having states adopt and enforce laws restricting commercial access with the ultimate goal of facilitating declines in tobacco usage. This study aimed to examine the impact of Synar policy implementation on minors’ commercial access to tobacco products in Mississippi over time, when compared with other Southern and non-Southern states in the U.S. The extremely low retailer violation rates (RVRs) in Mississippi make it an intriguing point of comparison with other states. Our investigation also examined patterns of minors’ non-commercial means for securing tobacco given more stringent controls on retail purchasing, while also considering overall youth patterns of tobacco use.

The central questions in our study led us to employ a before and after (pre-implementation and post-implementation) analytic strategy with a quasi-experimental design. The primary data source for this study was the Youth Risk Behavior Survey (YRBS), which was developed by CDC and administered by states. The quasi-control groups used in this study were YRBS respondents located in the South and the non-South. Mississippi was removed from both of these quasi-control groups so proper comparisons could be conducted. Using insights from a diffusion of health innovations perspective, the study examined temporal changes using repeated cross-sectional survey results from 1993 to 2011. Three time periods were analyzed: (1) pre-implementation, 1993–1997; (2) early post-implementation, 1999–2005; and (3) late post-implementation, 2007–2011. Binary logistic regression results supported Hypothesis 1, which predicted significant reductions in Mississippi youth’s commercial access to tobacco from pre-implementation to either post-implementation period. Results also supported a diffusion of innovation perspective for Mississippi youth (Hypothesis 2), such that reductions in access were significantly more pronounced in late post-implementation than early post-implementation. Hypothesis 3, which predicted greater reductions in young Mississippians’ commercial access to tobacco when compared with their Southern and non-Southern peers, given Mississippi’s especially low RVRs, was not supported. Finally, given Synar’s ultimate aim of reducing youth tobacco use and Mississippi’s especially aggressive approach to retail enforcement, Hypothesis 4 anticipated greater overall declines in minors’ tobacco use from 1993 to 2011 for Mississippi. This hypothesis was not supported. However, general declines in tobacco use were observed in all three study regions, including Mississippi, during this time. Consistent with insights from structuration theory, young Mississippians’ greater tendency to use non-commercial means to secure tobacco might account for the relatively smaller reductions in tobacco consumption among Mississippi youth across the study period.

There are a number of implications that stem directly from this study. First, this study illuminates debates over government-run drug control and prevention policies like Synar. Debates about Synar have raged since the policy was developed in 1992. Some of these debates initially hinged on the lack of research supporting the adoption of such a policy. In addition, many state administrators were displeased with Synar being imposed as an unfunded mandate that would cost states millions of dollars in block grant funds for non-compliance. While this study cannot adjudicate those elements of the controversy over this policy, the results presented here underscore the effectiveness of Synar in limiting youth access to tobacco and, as best as can be determined, contributing to declines in young people’s tobacco usage. So, on these fronts, there are indications that Synar has been a success. These results, then, could reduce some of the negative reactions toward Synar among states that, while criticizing the unfunded nature of the mandate, may recognize its ultimate utility. Given the emphasis placed on data-driven funding allocations, it may be time that effective tobacco control policies such as Synar were supported with steady funding streams.

Second, although Mississippi’s RVRs are considerably lower than those in the nation at large, this study has revealed that the effects of Synar in Mississippi are no more robust than those in other states. Thus, distinctive (and potentially costly) actions that Mississippi is taking to ensure low RVRs do not seem to be yielding stronger barriers to young people’s commercial access to tobacco or their use of tobacco products. Future research might explore different dependent variables, such as tobacco consumption based on an increasingly wide variety of tobacco products (vaping) or age of onset, that could be affected by Mississippi’s uniquely aggressive approach to retailer stings and lower RVRs. However, where commercial access and cigarette use are concerned, there is no clear dividend to be reaped from lower RVRs in our focal state. In fact, Mississippi youth were more inclined to pursue non-commercial means of securing tobacco after Synar implementation than youth in the rest of the South and in the remainder of the U.S.

Third, this study provides a broader lesson about the need to exhibit patience in determining the effectiveness of a newly implemented policy. If performance-based policymaking decisions are executed within a severely limited time frame, the most fruitful benefits of a newly adopted policy may not have had time to manifest themselves. In this sense, Synar is a good empirical case that supports the diffusion of health innovation framework and underscores the fact that “policy learning” is often a gradual process [35,37]. Furthermore, Synar is also a lesson in allowing the time necessary for an innovative policy to be implemented, refined, and then adjudicated as to its effectiveness. For a policy as complex as Synar, there were clearly years needed to perfect the retailer training and compliance system. Beyond the diffusion of innovation perspective that governed this study, we also drew insights from a structuration framework that highlights the limitations and unintended consequences of policy changes. The results of this study lend support to a structuration framework because our investigation revealed how policy gaps could be leveraged by some young Mississippians to create workarounds through which the intended outcomes of newly adopted policies are thwarted [31]. One such workaround that is more commonly used in Mississippi than elsewhere is youth enlisting non-commercial means to secure tobacco access.

Several limitations associated with our investigation are also worth noting, and these limitations suggest promising avenues for future research. First, our study was intentionally limited to contrast pre-Synar, early post-Synar, and late post-Synar implementation effects with periods of similar duration across each of these eras. Therefore, our study’s latest data year is some time ago (2011). A reasonable argument could be made for tracking even more recent implementation of the effects of Synar during the years following 2011. Where our investigation ended, others could certainly ensue. However, more recent YRBS data should be used with some caution, or perhaps paired with additional data sources, because some YRBS data waves are not consistently representative at the state level for all states in the U.S. Therefore, ending our study with the 2011 YRBS data year was intentional to create parity across study time periods, but also was necessitated by the changing contours and limitations of this data source in later years.

Second, new means for consuming tobacco have emerged in the past several years, and some of them (e.g., vaping) are particularly popular among young adults and youth. Such products, many of which have flavors designed to appeal to younger people, have altered the landscape of tobacco consumption in America. Therefore, researchers would do well to investigate how Synar fares with respect to newer means of consuming tobacco. Given that Mississippi youth used non-commercial means to secure tobacco for the duration of the post-Synar period investigated in our study, it seems plausible that youth vaping in Mississippi may be especially prevalent.

Finally, we have argued that Mississippi used an especially aggressive approach to Synar implementation by reviewing retailer violation rates (RVRs). However, we drew this conclusion from rates that, upon closer inspection, may not fully operationalize aggressive implementation. Researchers who work with rates realize that fluctuation in such statistics may occur for a number of reasons [8]. Rates can change based on variation in either the numerator or the denominator used to produce a rate. Thus, for example, especially low RVRs could result from more retailer stings, perhaps conducted less stringently if inspection capacity is limited (the denominator). Rates may also be lowered by fewer retail violations that indicate genuinely greater compliance on the part of commercial establishments (the numerator). So, some caution is needed in using rates as a key indicator of implementation. Moreover, how retail stings are conducted (e.g., certain months of the year or in a manner that permits retailers to alert one another about upcoming stings) could lead retailers to show more vigilance at some times than others. RVRs of this sort may be artificially deflated. Structuration theory provides a useful reminder that the maintenance of a social structure such as a law is subject to the routine social practices that are used to implement and enforce that law. RVRs provide an important window into routine implementation and enforcement practices, but state-run stings of retail establishments involve complex interactions between tobacco sellers, state inspectors, and the actual or would-be customers of those stores. Retailer violation rates also obscure important decisions about how, when, and where to conduct such stings, all of which could impact the resulting rates. If researchers are provided with more detailed information to generate RVRs, while being able to consider procedures for conducting stings, additional light could certainly be shed on implementation variations that remain unexplored in our study. We have conceded that various implementation mechanisms may undergird the relative effectiveness of Synar (increased strictness in state controls, enhanced capacity for retailer compliance) that we could not directly operationalize for this study. Nevertheless, additional research is needed to examine these implementation processes in tandem with outcomes. Until such research is conducted, the evidence presented here indicates that diligent prolonged action was needed to diffuse a large-scale policy innovation such as Synar effectively. Raising the legal age of tobacco product purchases to 21 is likely to reduce youth tobacco use and dependence further, and may be a more easily diffused innovation because of policy compliance practices initially instituted under Synar [6,7].

## 5. Conclusions

Passed by Congress in 1992, the Synar Amendment aimed to limit youth commercial access to tobacco products as a means of ultimately reducing minors’ consumption of tobacco. Previous research on Synar has revealed beneficial effects [6,7]. However, there has been a lack of attention to the comparison of state, regional, and national trends with a focus on diffusion effects. This paper adds to extant knowledge by contrasting tobacco access and tobacco use trends in three study domains: Mississippi, which has had an especially aggressive approach to Synar enforcement, was compared with the South and non-Southern states before and after Synar implementation. This study compared tobacco access before Synar (pre-implementation) and at two points afterward (early and late post-implementation). Trends in tobacco use for the duration of the study period were also compared. Results revealed that Mississippi’s Synar policy was effective, but not significantly more so than those evident in regional and national comparison groups. Moreover, our study indicated that Mississippi youth were more inclined than their peers elsewhere to secure tobacco through non-commercial means after the passage of Synar, thereby highlighting a significant limitation of policy implementation in this state. Further, and perhaps related to the elevated likelihood of Mississippi youth securing tobacco through non-commercial means, reductions in tobacco use among Mississippi youth were smaller in magnitude than their counterparts elsewhere in the U.S. The results of this study indicate that, while Synar has been broadly effective at reducing youth tobacco access and use, Mississippi’s especially aggressive approach to Synar enforcement has not created a distinctively effective anti-tobacco climate for youth in that state. In fact, because more successful outcomes have been observed throughout the U.S., youth tobacco prevention efforts in Mississippi may benefit from a careful examination of effective strategies pursued elsewhere in the country.

## Figures and Tables

**Table 1:** Retailer Violation Rates (RVRs) for Federal Fiscal Years (FFY) for 1997–2011.

FFY	‘97	‘98	‘99	‘00	‘01	‘02	‘03	‘04	‘05	‘06	‘07	‘08	‘09	‘10	‘11
MS	40.0	30.5	32.7	29.9	11.6	5.7	7.7	3.9	6.0	3.2	3.2	5.1	3.8	3.8	2.3
South	29.4	25.4	17.7	19.1	16.6	14.7	12.8	12.9	12.1	10.9	9.6	10.5	11.1	11.1	9.8
Non-South	36.9	23.4	21.8	20.9	18.0	17.5	14.9	12.9	12.1	10.9	11.0	9.7	10.9	8.4	7.9

Note: RVRs are weighted by population. South consists of AL, AK, DE, DC, FL GA, KY, LA, MD, NC, OK, SC, TN, TX, VA, and WV. Sources: U.S. Department of Health & Human Services [23]; U.S. Census Bureau [24,25].

**Table 2:** Trends in adjusted proportion of commercial access.

Study Region	Pre-Synar			Early Post-Synar				Late Post-Synar			Change
	1993	1995	1997	1999	2001	2003	2005	2007	2009	2011	
**Mississippi**											
Adjusted% ^a^	-	18.66	11.6	10.42	6.74	7.04	-	6.02	6.09	5.75	−69%
N	-	1267	1528	1552	1797	1478	-	1597	1788	1820	
**South**											
Adjusted% ^a^	-	16.83	17.39	12.48	9.36	7.22	7.39	6.71	5.68	6.21	−63%
N	-	4628	6230	7456	5892	7999	7049	5725	5322	6429	
**Non-South**											
Adjusted% ^a^	-	16.63	13.09	11.25	8.38	5.99	5.25	4.79	4.84	4.66	−73%
N	-	5378	9675	7159	7305	7134	6749	7879	10,986	8812	

^a^ Means are adjusted for age, gender, and race-ethnicity. Design effects are corrected with robust standard errors and weights. Log[*p*(x)/1−*p*(x)] = *α* + *β*_1_Age + *β*_2_Male + *β*_3_Black + *β*_4_Hispanic + *β*_5_OtherRace.

**Table 3:** Quasi-Experimental Effects of Synar on Commercial Access: 1993–2011 ^a^.

Variables	Mississippi		South		Non-South	
Age	1.911	0.000	2.190	0.000	1.997	0.000
Male ^b^	1.893	0.000	1.566	0.000	1.385	0.000
Black	0.351	0.000	0.335	0.000	0.628	0.001
Latino	1.009	0.966	0.716	0.000	0.685	0.000
Other race ^c^	0.840	0.404	0.661	0.004	0.816	0.047
Early Post-Synar Era (1999–2005) ^d^	0.474	0.000	0.496	0.000	0.524	0.000
Late Post-Synar Era (2007–2011) ^d^	0.319	0.000	0.322	0.000	0.307	0.000
Constant	0.006	0.001	0.003	0.001	0.004	0.001
N	12,244		52,613		66,643	

^a^ Binary logistic regression odds coefficients are displayed with associated *p* values. ^b^ Female is reference. ^c^ White is reference. ^d^ Pre-Synar is reference. Note: Design effects are corrected with robust standard errors and weights. Log[*p*(x)/1 − *p*(x)] = *α* + *β*_1_Age + *β*_2_Male + *β*_3_Black + *β*_4_Hispanic + *β*_5_OtherRace + *β*_6_Early Post-Synar + *β*_7_ Late Post-Synar.

**Table 4:** Interaction Effects of Synar Era and Region ^a^.

Variables	Odds Coefficients	Significance
Age	1.925	0.018
Male ^b^	1.841	0.022
Black	0.352	0.075
Latino	0.812	0.083
Other race ^c^	0.824	0.117
Early Post Synar Era (1999–2005) ^d^	0.473	0.043
Late Post Synar Era (2007–2011) ^d^	0.319	0.008
South ^e^	0.840	0.109
Non-South ^e^	0.656	0.091
Early Post Synar Era × South	1.057	0.124
Early Post Synar × Non-South	1.105	0.120
Late Post Synar Era × South	1.024	0.120
Late Post Synar × Non-South	0.950	0.099
Constant	0.006	0.107
N	131,500	

^a^ Binary logistic regression odds coefficients are displayed with associated *p* values (significance). ^b^ Female is reference. ^c^ White is reference. ^d^ Pre-Synar is reference. ^e^ Mississippi is reference. Design effects are corrected with robust standard errors and weights.

**Table 5:** Quasi-Experimental Effects of Synar on Non-Commercial Access: 1993–2011 ^a^.

Variables	Mississippi		South		Non-South	
Age	0.949	0.161	0.945	0.008	0.941	0.006
Male ^b^	1.065	0.010	0.845	0.004	0.706	0.000
Black	0.367	0.000	0.321	0.000	0.369	0.000
Latino	0.819	0.504	0.599	0.000	0.745	0.000
Other race ^c^	0.899	0.093	0.802	0.104	0.863	0.165
Early Post-Synar Era (1999–2005) ^d^	1.428	0.007	1.353	0.015	1.024	0.800
Late Post-Synar Era (2007–2011) ^d^	1.476	0.033	1.002	0.984	0.704	0.000
Constant	0.128	0.001	0.147	0.000	0.147	0.000
N	12,254		52,613		66,643	

^a^ Binary logistic regression odds coefficients are displayed with associated *p* values. ^b^ Female is reference. ^c^ White is reference. ^d^ Pre-Synar is reference. Design effects are corrected with robust standard errors and weights. Log[*p*(x)/1 − *p*(x)] = *α* + *β*_1_Age + *β*_2_Male + *β*_3_Black + *β*_4_Hispanic + *β*_5_OtherRace + *β*_6_Early Post-Synar + *β*_7_ Late Post-Synar.

**Table 6:** Trends in Adjusted Average 30-day Use (1 = 0 days, …, 7 = all 30 days).

Mississippi	1993	1995	1997	1999	2001	2003	2005	2007	2009	2011	Change
Adjusted Mean ^a^	2.07	2.25	2.20	2.20	1.91	1.97	-	1.67	1.71	1.64	−21%
N	1391	1267	1528	1552	1797	1478	-	1597	1788	1820	
**South**											
Adjusted Mean ^a^	2.17	2.22	2.54	2.47	2.21	1.87	1.99	1.91	1.76	1.64	−24%
N	5142	4628	6230	7456	5892	7999	7049	5725	5322	6429	
**Non-south**											
Adjusted Mean ^a^	2.14	2.37	2.28	2.26	1.99	1.80	1.71	1.59	1.62	1.57	−27%
N	7412	5738	9675	7159	7305	7134	6749	7879	10,986	8812	

^a^ Means are adjusted for age, gender, and race-ethnicity. Design effects are corrected with robust standard errors and weights.
